# Bacteriophage therapy for multidrug-resistant pediatric infections: a re-emerging precision antimicrobial strategy

**DOI:** 10.1097/MS9.0000000000005020

**Published:** 2026-04-15

**Authors:** Asra Amjad, Armeen Shahzad, Fred Segawa

**Affiliations:** a Department of Medicine, Islamic International Medical College, Riphah International University, Rawalpindi, Pakistan; b Department of Medicine, Dow Medical College, Dow University of Health Sciences, Karachi, Pakistan; c Department of Medicine, College of Health Sciences, Makerere University, Kampala, Central Region, Uganda

*To the Editor,*

The urgent need for innovative medicines is highlighted by the rising ineffectiveness of current antibiotics in children, particularly in neonatal intensive care units, due to antimicrobial resistance. Alternative methods that are accurate and successful are currently being re-examined in light of this pressing requirement. Using viruses that infect and destroy particular bacteria, bacteriophage therapy provides targeted antimicrobial treatment^[^1^]^. The most remarkable aspect of the treatment is the phages’ selectivity, which enables them to target just pathogenic bacteria while sparing the body’s beneficial bacteria. This is an essential aspect of the treatment, particularly for youngsters^[^2^]^. Innovations in laboratory methods that enable medical professionals to pinpoint the precise bacterial strains causing infection further improve this accuracy. The advancements in microbiological testing and genetic sequence analysis allow us to create a customized phage therapy cocktail based on the specific bacterial infections found in a patient. This is an example of managing infectious infections using precision medicine^[^3^]^. Phage treatment has promise in treating children with infections that are difficult to treat, such as *Pseudomonas aeruginosa* lung illness in cystic fibrosis, multi-resistant sepsis in infants, and chronic osteomyelitis. Case studies of compassionate use have shown that bacterial illnesses resistant to conventional antibiotic therapy can be cleared^[^4,5^]^. Beyond individual cases, there is mounting evidence that a more effective approach to combating resistant illnesses may involve combining phages with currently available medicines. There is mounting evidence that phages and antibiotics may work in concert to boost antibacterial effectiveness and possibly delay the development of resistance^[^2^]^. Phage therapy looks to be safe based on data from clinical research thus far, but it is crucial to keep an eye on the immune response to phage infection and the endotoxin release that follows bacterial lysis^[^6,7^]^. Despite these promising outcomes, there are still major manufacturing and regulatory obstacles to overcome before phage therapy may be routinely used in pediatric care. However, there are several obstacles to its actual use despite its encouraging therapeutic promise. Standardizing phage production to target certain bacteria is a complicated problem in terms of existing pharmaceutical rules, and the regulatory system is still in its infancy. However, current advances in synthetic biology and the application of CRISPR technology are providing new opportunities for modified phages with increased antibacterial activity to more successfully target biofilms and resistance genes^[^3^]^. When considered collectively, these advancements indicate that bacteriophage therapy has the potential to be a useful supplement to traditional antibiotics. A tailored antimicrobial strategy employing bacteriophage therapy could supplement conventional antibiotics and boost treatment options for kids sick with multidrug-resistant bacteria as antimicrobial resistance rises globally^[^7–9^]^.

In summary, bacteriophage therapy is a child-friendly, precision-based approach that may help close the increasing gap left by ineffective antibiotics, providing new hope for the treatment of pediatric infections resistant to several drugs and opening the door to individualized antimicrobial care.

TITAN Guidelines: This manuscript is in compliance with TITAN Guidelines 2025 declaring no use of AI**^[^10^]^.**

## Data Availability

Not applicable.
